# Flow cytometry quantification of tumor-infiltrating lymphocytes to predict the survival of patients with diffuse large B-cell lymphoma

**DOI:** 10.3389/fimmu.2024.1335689

**Published:** 2024-01-29

**Authors:** Tiantian Yu, Zijun Y. Xu-Monette, Anand Lagoo, Wen Shuai, Bangchen Wang, Jadee Neff, Luis F. Carrillo, Eric D. Carlsen, Sergio Pina-Oviedo, Ken H. Young

**Affiliations:** ^1^Hematopathology Division and Department of Pathology, Duke University Medical Center, Durham, NC, United States; ^2^Duke University Cancer Institute, Durham, NC, United States; ^3^Department of Pathology, Duke University Medical Center, Durham, NC, United States

**Keywords:** tumor-infiltrating lymphocytes, normal B cells, TIL, DLBCL, prognosis, microenvironment, flow cytometry, single-cell

## Abstract

**Introduction:**

Our previous studies have demonstrated that tumor-infiltrating lymphocytes (TILs), including normal B cells, T cells, and natural killer (NK) cells, in diffuse large B-cell lymphoma (DLBCL) have a significantly favorable impact on the clinical outcomes of patients treated with standard chemoimmunotherapy. In this study, to gain a full overview of the tumor immune microenvironment (TIME), we assembled a flow cytometry cohort of 102 patients diagnosed with DLBCL at the Duke University Medical Center.

**Methods:**

We collected diagnostic flow cytometry data, including the proportion of T cells, abnormal B cells, normal B cells, plasma cells, NK cells, monocytes, and granulocytes in fresh biopsy tissues at clinical presentation, and analyzed the correlations with patient survival and between different cell populations.

**Results:**

We found that low T cell percentages in all viable cells and low ratios of T cells to abnormal B cells correlated with significantly poorer survival, whereas higher percentages of normal B cells among total B cells (or high ratios of normal B cells to abnormal B cells) and high percentages of NK cells among all viable cells correlated with significantly better survival in patients with DLBCL. After excluding a small number of patients with low T cell percentages, the normal B cell percentage among all B cells, but not T cell percentage among all cells, continued to show a remarkable prognostic effect. Data showed significant positive correlations between T cells and normal B cells, and between granulocytes and monocytes. Furthermore, we constructed a prognostic model based on clinical and flow cytometry factors, which divided the DLBCL cohort into two equal groups with remarkable differences in patient survival and treatment response.

**Summary:**

TILs, including normal B cells, T cells, and NK cells, are associated with favorable clinical outcomes in DLBCL, and flow cytometry capable of quantifying the TIME may have additional clinical utility for prognostication.

## Introduction

Diffuse large B-cell lymphoma (DLBCL) is a mature B-cell lymphoma with aggressive clinical and biological characteristics (1). With the standard immunochemotherapy rituximab (R, a CD20-targeting monoclonal antibody) in combination with cyclophosphamide, doxorubicin, vincristine and prednisone (CHOP), approximately 40% of patients with DLBCL experience treatment failure and are not cured (2). In the past two decades, a great deal of effort has been devoted to improving the clinical outcome of R-CHOP as the frontline treatment for DLBCL, including shortening the interval between treatment cycles, intensifying the treatment, or incorporating new drugs alongside R-CHOP. However, these efforts have not yielded successful results (2–4).

Immunotherapeutic approaches hold promise in lymphoma treatment, as demonstrated by the great success of chimeric antigen receptor (CAR) T cell therapies in relapsed/refractory DLBCL and the approval of programmed cell death protein 1 (PD-1) blockade in relapsed/refractory Hodgkin lymphoma and primary mediastinal large B-cell lymphoma (5–9). “Hot” tumors, characterized by a high presence of tumor-infiltrating lymphocytes (TILs), demonstrate a markedly improved response to checkpoint inhibitors (10–12). However, the majority of DLBCLs are either “cold” or have multiple immune escape and immunosuppressive mechanisms, which could contribute to the less-than-optimal outcomes of anti-PD-1 and CAR-T cell immunotherapies in these cases (13–15). The tumor immune microenvironment (TIME) in DLBCL is highly intricate and heterogeneous in DLBCLs, with reciprocal interactions among tumor cells, adaptive and innate immune cells, their soluble mediators, and structural components within the tumor microenvironment (TME), which plays an important role in regulating tumor initiation, progression, and metastasis (16, 17). In our previous study implementing fluorescent multiplex immunohistochemistry (mIHC) in a large cohort of patients with DLBCL treated with R-CHOP, we found that T cell deficiency, natural killer (NK) cell deficiency, high percentage expression of PD-1 in CD8^+^ T cells, and PD-L1 expression in the TIME were associated with significantly poorer survival (18). Moreover, by ultra-deep sequencing of immunoglobulin genes, we found that higher frequencies of normal B cells (distinguished by clonotype analysis) in total B cells were associated with significantly better survival in DLBCL (19).

DLBCLs are characterized by a dense population of tumor B cells that obscure normal tissues and hinder the identification of non-malignant cell types such as immune and stromal cells within the TME (20). Flow cytometry is a single-cell technique that separates heterogeneous clonal cell populations in a mixed suspension based on cellular complexity and size. It can swiftly and quantitatively assess the expression of multiple markers at the single-cell level, and is therefore routinely used in the clinical diagnosis of hematologic malignancies (21, 22). Flow cytometry is capable of measuring six to fifteen parameters at high flow rates (>20,000 cells per second) (23, 24). In this study, we retrospectively analyzed the flow cytometry data of tumor tissues from 102 patients diagnosed with DLBCL for the prognostic impact of heterogeneous TIME components in the assembled cohort.

## Methods

### Patients

This study was approved as exempt by the Institutional Review Board of Duke University and conducted in accordance with the principles of the Declaration of Helsinki. We collected clinical information and flow cytometry results of diagnostic lymph node samples from 102 patients with DLBCL diagnosed at Duke University Hospital between 2011 and 2022, confirmed by morphology and immunohistochemistry (IHC) results according to the World Health Organization classification (25). DLBCL cases transformed from indolent lymphoma, post-transplant lymphoproliferative disorder, or HIV-related DLBCL cases were not included. The final cohort included 84 patients with DLBCL, not otherwise specified, nine patients with primary mediastinal DLBCL, two patients with primary brain DLBCL, one patient with primary DLBCL of the lacrimal gland, one patient with primary testicular lymphoma (PTL), two patients with primary cutaneous lymphoma, and three patients with DLBCL/high-grade B-cell lymphoma with *MYC* and *BCL2* rearrangements (26).

The study cohort had a mean age of 62.3 years old, and 53.9% were male. The median follow-up duration was 36.8 months. At the last follow-up, 66 patients were alive and 36 patients had died. 92 patients received R-containing immunochemotherapy at the frontline, including R-CHOP, n=58; R-CHOP and R-EPOCH, n=3; R-EPOCH, n=23; R-CEOP, n=2; hyperCVAD and R-CHOP/EPOCH-R, n=2; bendamustine-R, n=2; rituximab, temozolomide, and methotrexate regimen, n=2. In addition, one patient was treated with hyperCVAD plus MTX and cytarabine, one patient received CHOP without R, one patient who was not a candidate for systemic chemotherapy due to medical comorbidities received only radiotherapy for DLBCL (survival, 5.7 months), six patients did not receive treatment for DLBCL due to poor health condition (four of these patients died within a month, and the other two patients died in less than 2 months after diagnosis), and one patient with DLBCL diagnosed after small bowel resection chose no chemotherapy and then received standard R-CHOP treatment at recurrence (at 24 months after diagnosis). Ten patients with refractory (n=3) or relapsed (n=7) disease received CAR-T therapy. One patient who received CAR-T therapy after failed R2 and nivolumab treatments died of cholelithiasis and sepsis after severe and prolonged neurotoxicity and complications associated with cytokine release syndromes, and one of these 10 patients has died of relapse.

### Flow cytometry analysis

Fresh lymphoma tissue fragments (≥0.1×0.1 cm) were disaggregated to release cells, which were then washed, centrifuged, and the cell pellets were re-suspended in PBS/0.1% azide. Fine needle aspirates were used for 11 patients (four of these patients also had tissue fragments for flow cytometry analysis). The generated cell suspensions were adjusted to 0.5- 1× 10^6^ cells per tube, incubated with fluorochrome-conjugated antibody cocktails in the dark at room temperature for 15 minutes and followed the “stain/lyse/fix” wash protocol as described previously (27, 28). According to Duke Clinical Flow Cytometry Laboratory Standard of Operating Procedure, antibodies were conjugated to fluorescein isothiocyanate (FITC), phycoerythrin (PE), peridinin chlorophyll protein (PerCP) cyanine 5.5, PE-Cy7, allophycocyanin (APC), APC-H7, V450, or V500 (8 fluorochromes after 2014), including V500 anti-human CD45, PerCP-Cy5.5 anti-human CD19, PE-Cy7 anti-human CD5, APC-H7 anti-human CD3, PE anti-human CD7, APC anti-human CD10, APC-H7 anti-human CD20, PE anti-human lambda, FITC anti-human kappa (eBioscience, Inc., USA), and other antibodies against B/NK/myeloid/stem cell antigens. Various combinations were used to form B cell/T cell/Myeloid/stem cell/miscellaneous workup panels [8-multicolor panels for most patients (29)] to analyze different tubes of cell suspensions and controls. Stained cells were acquired on a Fortessa flow cytometer (dual laser FACSCanto II or FACSCalibur, Becton Dickinson Biosciences, USA) and analyzed using the Cellquest Becton Dickinson Biosciences) or FCS Express (de Novo Software, Glendale, CA) analysis software programs. The analysis consists of eliminating debris and gating on the lymphocytes. Quadrant markers in biparametric dot plots are adjusted for the negative and positive populations, so that lymphocytes stained by the antibodies are easily identified and distinctly separated into specific subpopulations.

### Statistical analysis

Statistical analyses were performed using R software (RRID : SCR_001905) for Windows (version 4.2.3, https://www.r-project.org/), GraphPad Prism version 9 (RRID : SCR_002798), and IBM SPSS (RRID : SCR_016479). Continuous variables are shown as mean ± SD or median, and categorical variables are shown as n (%). Correlations between immune cell types were evaluated using the Pearson’s correlation coefficient. Kaplan-Meier survival curves and log-rank tests were used to analyze the differences in overall survival (OS) and progression-free survival (PFS) calculated from date of diagnosis between the two groups. X-tile software (version 3.6.1, Yale School of Medicine, New Haven, CT) was used to determine the optimal cut-off values for immune cell data to predict OS. The Cox regression method in SPSS was used for the multivariate analysis. Alternatively, factors with statistical significance in univariate analysis were subjected to LASSO-Cox regression analysis for dimensionality reduction. The optimal penalty value lambda in the LASSO regression was first obtained using cross-validation, a Cox multivariate regression model was then established, and a nomogram was built using R software. The discrimination and accuracy of the prediction model were evaluated using time-dependent receiver operating characteristics (ROC) curves, calibration curves, and decision curve analysis. The risk score for each patient was calculated based on the LASSO-Cox prediction model. All patients were then divided into two groups according to the risk scores. All reported *P*-values were two-tailed, and statistical significance was set at *P* < 0.05.

## Results

### TIME characteristics determined by flow cytometry

**Figure 1:**
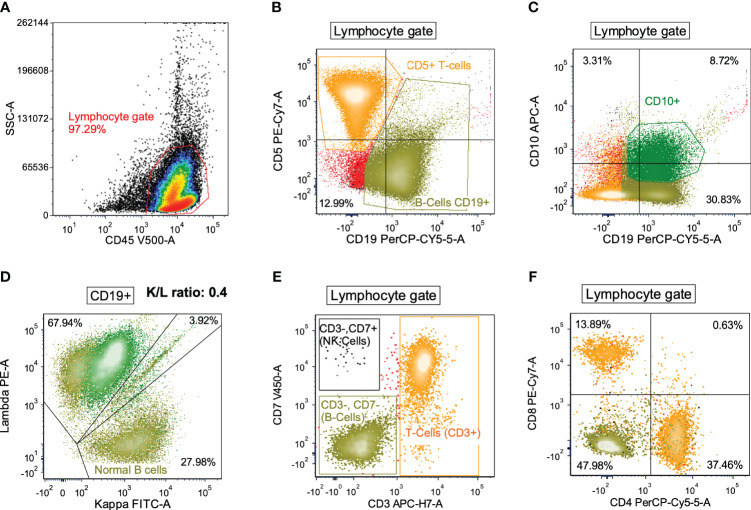
Flow cytometry dot plots for a representative DLBCL case with high proportions of T cells and normal B cells and low proportions of NK cells, monocytes, and granulocytes. **(A)** All viable cells (as determined by FSC and SSC, not shown) were evaluated by CD45 positivity and SSC (lymphocyte gate). **(B)** Lymphocytes were then differentiated by CD5 and CD19 where T-cells (orange) and B-cells (olive green) were distinguished. **(C)** Lymphocytes were also differentiated by CD10 and CD19 expression in order to isolate the CD10+/CD19+ B-cells (dark green) from the CD10-negative/CD19+ B-cells (olive green). **(D)** B-cells were then evaluated for kappa and lambda immunoglobulin light chain expression. Overall the K/L ratio was 0.4:1, but distinction between CD10+ and CD10-negative B-cells showed that the CD10+ B-cells were lambda-restricted (dark green) while the CD10-negative B-cells were polytypic (olive green). **(E, F)** Lymphocytes were also evaluated for CD3 and CD7 expression to distinguish T-cells (orange), B-cells (olive green), and NK-cells (black) (panel **E**). T-cells showed a normal CD4:CD8 ratio (panel **F**).

From the *de novo* DLBCL cases diagnosed between 2011 and 2022 at the Duke University Medical Center, we selected cases with diagnostic flow cytometry data of abnormal B cells and immune cells. A total of 102 DLBCL patients with available clinical and flow cytometry data were included in this study. Cohort characteristics are shown in **Table 1**. In flow cytometry study viable cells were selected by forward and side light scatter (FSC and SSC), and lymphocytes, granulocytes and monocytes were first distinguished by CD45+ and SSC, and then examined by markers in myeloid panels. Granulocytes were identified based on dim CD45 and high SSC values. Next, lymphocytes were differentiated by expression of surface markers in the multicolor panels. CD19-negative CD5+ (performed in 102 cases), CD3+CD4+, and CD3+CD8+ (performed in 81 cases) cell clusters were identified as T cells, CD19+, CD20+, CD10-negative/+, CD5-negative/+ cell clusters with kappa/lambda light chain restriction were identified as abnormal (malignant) B cells (CD20-negative abnormal B cells (30) were identified in four cases), CD38-bright CD20-negative CD19-positive cells were identified as normal or abnormal plasma cells, and NK cells were identified from CD3-negative CD7+ and CD3-negative CD56+ cell clusters. **Figure 1** shows flow cytometry dot plots for a representative case. Abnormal B cells were isolated for diagnosis by gating using various marker combinations and monotypic lambda light chain expression. Polyclonal kappa/lambda light chain-positive CD19+ CD20+ CD10-negative CD5-negative cell clusters were identified as normal (non-malignant) B cells, whose percentages was quantified and reported (**Supplementary Figure 1A**) along with other separated cell clusters. T cells were identified by either CD3 or CD5 gating on lymphocytes, and further subtyped by CD4 and CD8 markers. A CD3 vs. CD7 plot distinguished T cells, B-cells (CD3-negative CD7-negative) and NK-cells (CD3-negative CD7+).

**Table 1:** Clinical characteristics of the patients with DLBCL in the study cohort and tumor tissue constitutions as determined by flow cytometry.

Characteristic	Results in the cohort
Age >60 years, n (%)	62 (60.8)
Sex: Male, n (%)	55 (54)
Female, n (%)	47 (46)
Ann Arbor stage III or IV, n (%)	50 (49.0)
LDH >ULN, n (%)	66 (66.0)
ECOG performance status ≥2, n (%)	18 (17.6)
Extranodal sites ≥2, n (%)	20 (19.6)
IPI >2, n (%)	39 (38.27)
B symptoms present, n (%)	49 (48.0)
Mass size ≥7.5cm, n (%)	33 (32.4)
None-GC subtype, n (%)	48 (47.1)
Race: Caucasian	84 (82.4)
Black	13 (12.7)
Hispanic, Asian	5 (4.9)
Treatment response: CR, n (%)	60 (75)
PR, n (%)	12 (15)
PD, n (%)	8 (10)
CD5+ T% in all viable cells, mean ± SD, median	41.0 ± 22.4, 42.75
Abnormal B % in all viable cells, mean ± SD, median	39.06± 27.19, 36.74
Granulocytes % in all cells, mean ± SD, median	6.10 ± 11.87, 1.34
Normal B % in all viable cells, mean ± SD, median	4.03 ± 5.65, 2.08
NK % in all cells, mean ± SD, media	1.08 ± 1.11, 0.81
Monocytes % in all cells, mean ± SD, median	1.18 ± 2.22, 0
T (CD5):Abnormal B ratio, mean ± SD, median	4.17 ± 10.10, 1.25
Normal B:Abnormal B ratio, mean ± SD, median	0.35 ± 0.77, 0.048
Normal B % in all B cells, mean ± SD, median	15.16 ± 21.25, 4.60

LDH, lactate dehydrogenase; ULN, upper limit of normal; ECOG, Eastern Cooperative Oncology Group; IPI, International Prognostic Index; GCB, germinal center B-cell; CR, complete remission; PR, partial response; PD, progression; SD, standard deviation.

**Figure 2:**
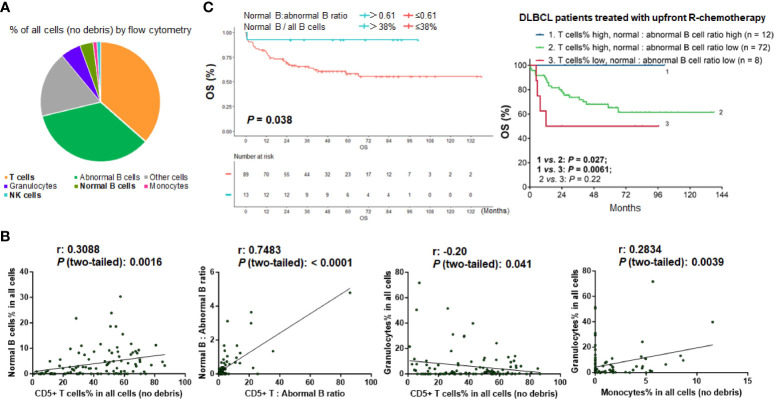
Analysis for the TIME components in the study cohort. **(A)** Pie chart showing the mean proportion of cell clusters in the DLBCL cohort. **(B)** Pearson correlation analysis. T cells showed highly significant positive correlation with normal B cells and weak negative correlation with granulocytes. Granulocytes showed significant positive correlation with monocytes (by percentage in all viable cells). Each dot in the scatter plots shows the flow cytometry result for one patient. **(C)** Patients with high ratios of normal B cells to abnormal B cells (that is, patients with high percentage of normal B cells in all B cells) had significant better survival in the overall cohort and in patients with high percentages of T cells in all viable cells.

The mean proportion of each immune cell type among all cells (no debris) in the study cohort was 41.0% for T cells, 4.0% for non-malignant B cells, 1.1% for NK cells, 1.2% for monocytes, and 6.1% for granulocytes (**Figure 2A**). The median proportions of T cell, normal B cells, NK cells, monocytes, and granulocytes among all cells were 42.75%, 2.08%, 0.81%, 0%, and 1.34%, respectively (**Table 1**). Plasma cells were rare and only found in 12 patients at a low percentage (only two patients had plasma cells accounting for >1% of all cells), with a median of 0.41% and a mean of 0.85%.

Correlations between various types of immune cells were also analyzed. The percentage of T cells was significantly correlated with the percentage of normal B cells (r = 0.31, *P* = 0.0016). The percentage of granulocytes was significantly correlated with the percentage of monocytes (r = 0.28, *P* = 0.0039). In addition, there was a weak negative association between granulocyte and T cell percentages (r = -0.20, *P* = 0.041) (**Figure 2B**). The matrix of the association results is shown in **Supplementary Figure 1B**.

Moreover, we evaluated the abundance of immune cells (T cells, normal B-lymphocytes [including plasma cells], NK cells, monocytes, and granulocytes) relative to tumor cells by calculating the ratios of immune cells to abnormal B cells in flow cytometry reports for each case. The results for T cells and normal B cells were significantly correlated (r = 0.75, *P* < 0.0001; **Figure 2B**). The mean ratios of T cells and normal B cells to abnormal B cells were 4.2 and 0.35, respectively; the median ratios were 1.25 and 0.048 (4.6% of all B cells), respectively.

### Biomarker identification by univariate analysis

**Figure 3:**
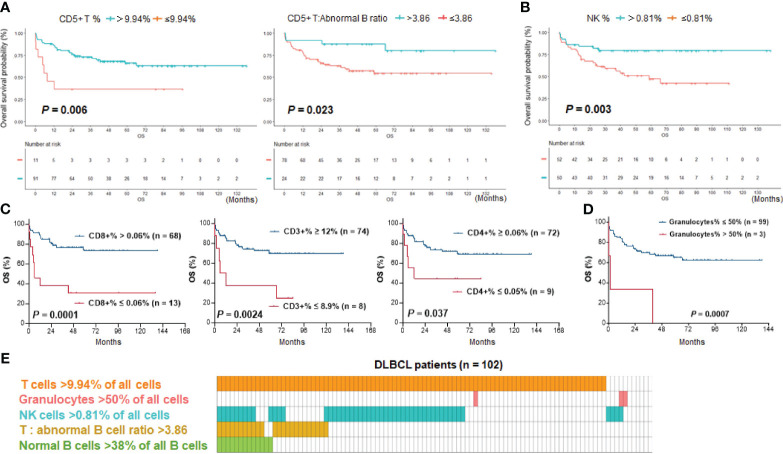
Kaplan–Meier survival analysis. **(A)** T cell abundance, either by percentage of CD5+ T cells in all cells or by ratio of CD5+ T cells to abnormal B cells, was associated with significantly better survival of DLBCL patients. **(B)** High percentages of NK cells in all cells were associated with significantly better survival of patients. **(C)** High percentages of CD3+, CD4+, and CD8+ T cells in all cells were associated with significantly better survival of DLBCL patients with data available. The effect by CD8+ T cells was most significant. **(D)** Three DLBCL cases with a high percentage of granulocytes in all cells (>50%) had significantly poorer survival. **(E)** Distribution plot illustrating patient groups stratified by immune cell proportions with significant prognostic differences in the study cohort. One column represents one patient.

After characterizing the TIME constitution for each case, we used the Kaplan-Meier method to analyze the prognostic effects of immune cell abundance in the DLBCL cohort, including T cells, normal B cells, NK cells, monocytes, and granulocytes evaluated by their percentages in all cells or by their ratios to abnormal cells. We found that only high ratios of normal B cells to abnormal B cells (>0.61, equivalent to 38% of all B cells, **Figure 2C**), high ratios of T cells to abnormal B cells (>3.86), high percentages of T cells among all cells (≥9.94% using CD5 marker and 12% using CD3 marker in the evaluated cases), and high percentages of NK cells (>0.81%) among all cells were associated with significantly better OS of patients (**Figures 3A-C**). In contrast, very high percentages (>50%) of granulocytes were associated with significantly worse OS (**Figure 3D**); however, the granulocytes-high group had only three patients. In the high T cell-percentage group, T cell percentages in all cells did not show further prognostic effects (not significant with all cutoffs shown in the X-tile analysis); however, high ratios of T cells to abnormal B cells (>3.86) were associated with significantly better OS (**Supplementary Figure 1C**). High ratios of normal B cells to abnormal B cells (and high percentages of normal B cells among all B cells) showed an even more remarkably superior OS among patients with high T cell percentages. We further verified these significant prognostic effects in treated patients (in fact, normal B cells showed more significant prognostic effect in treated patients. **Supplementary Figure 2A**; **Figure 2C**). **Figure 3E** illustrates the distribution of patient groups stratified by the TIME factors with prognostic significance.

Kaplan-Meier survival analysis was also performed for clinical parameters, germinal center (GC)/non-GC subtypes, and treatment factors (**Table 2**). Age, performance status, elevated serum lactate dehydrogenase (LDH) level, and presence of B symptoms were significant prognostic clinical factors in this cohort, and advanced disease stage showed border-line significance of adverse prognostic impact. Among patients treated with upfront systemic chemotherapy, those who received R-CHOP had significantly better OS than those who received only non-R-CHOP regimens. Transplantation, but not radiotherapy, was associated with significantly better OS. The favorable impact of CAR-T therapy (in only ten patients) was not significant in the entire cohort but was significant in relapsed/refractory patients (**Supplementary Figures 2B–D**).

**Table 2:** Kaplan-Meier Log-rank (Mantel-Cox) analysis for overall survival in the study cohort.

Factor	Prognostic effect	Percentage	*P*
Age >60	Unfavorable	62/102, 61%	**0.001**
Male	Favorable	55/102, 54%	0.25
Stage 3/4	Unfavorable	50/102, 49%	**0.051**
LDH elevated	Unfavorable	66/100, 66%	**0.02**
ECOG performance status >1	Unfavorable	18/102, 18%	**<0.0001**
# of extranodal sites >1	Unfavorable	20/102, 20%	0.10
B symptoms	Unfavorable	49/102, 48%	**0.003**
Largest tumor size ≥7.5cm	Unfavorable	33/102, 32%	0.87
IPI >2	Unfavorable	39/102, 38%	**<0.0001**
Race, white		84/102, 82%	0.93
Non-GC subtype	Unfavorable	48/102, 47%	0.24
T cell (CD5+) percentage of all cells >9.94%	Favorable	91/102, 89%	**0.006**
T to abnormal B cell ratio >3.86	Favorable	24/102, 24%	**0.023**
B to abnormal B cell ratio >0.61	Favorable	13/102, 13%	**0.038**
NK cell percentage in all cells >0.81%	Favorable	50/102, 49%	**0.003**
Granulocyte percentage in all cells >50%	Unfavorable	3/102, 3%	**0.001**
T cell (CD3+) percentage in all cells ≥12%	Favorable	74/82, 90%	**0.003**
CD4+ cell percentage in all cells ≥0.06%	Favorable	72/81, 89%	**0.04**
CD8+ cell percentage in all cells >0.06%	Favorable	68/81, 84%	**0.0001**
R-CHOP in treated patients	Favorable	63/96, 66%	**0.02**
R-CHOP in upfront curative-intent chemotherapies	Favorable	63/94, 67%	**0.026**
Transplantation	Favorable	12/92, 13%	**0.037**
CAR-T therapy in overall cohort	Favorable	10/102, 9.8%	0.30
CAR-T therapy in relapsed/refractory patients	Favorable	10/49, 20.4%	**0.0056**

LDH, lactate dehydrogenase; ECOG, Eastern Cooperative Oncology Group; IPI, International Prognostic Index; GC, germinal center; R-CHOP, rituximab, cyclophosphamide, doxorubicin, vincristine and prednisone; CAR-T, chimeric antigen receptor T cell. Significant *P* values are in bold.

### Multivariate analysis and prognosis prediction

Two methods were used for multivariate analysis of OS. First, a Cox regression (proportional hazards) model that incorporated all parameters was used in SPSS software to analyze the OS in the study cohort. The results (**Table 3**) showed that the T cell percentage in all cells, ratio of normal B cells to abnormal B cells (or percentage of normal B cells in all B cells), age, performance status, number of extranodal sites, presence of B-symptoms, race, and receiving CAR-T therapy were significant prognostic factors.

**Table 3:** Multivariate overall survival analysis by Cox regression (proportional hazards) in the study cohort.

Factor	Hazard ratio	95% CI	*P*
Age >60	16.7	4.06-59.1	**<0.001**
Male	0.58	0.22-1.51	0.26
Stage 3/4	0.64	0.19-2.2	0.48
LDH elevated	0.84	0.23-3.03	0.79
ECOG performance status >1	14.2	4.79-42.3	**<0.001**
# of extranodal sites >1	3.69	1.11-12.3	**0.033**
B symptoms	4.04	1.53-10.7	**0.005**
Largest tumor size ≥7.5cm	1.77	0.58-5.38	0.33
Race, white	0.31	0.096-0.97	**0.044**
Non-GC subtype	0.91	0.35-2.39	0.85
T cells, % of all cells	0.34	0.12-0.94	**0.038**
T to abnormal B cell ratio >3.86	0.70	0.13-3.79	0.68
Normal B to abnormal B cell ratio >0.61	0.044	0.004-0.51	**0.012**
NK cells, % of all cells >0.81%	0.51	0.17-1.50	0.22
Granulocytes, % of all cells >50%	1.14	0.20-6.47	0.88
Treatment and frontline regimens	0.71	0.39-1.30	0.27
Transplantation	5.62	0.43-73.4	0.19
CAR-T therapy	0.031	0.002-0.53	**0.016**

CI, confidence interval; LDH, lactate dehydrogenase; ECOG, Eastern Cooperative Oncology Group; GC, germinal center; CAR, chimeric antigen receptor. Significant *P* values are in bold.

Second, LASSO-Cox analysis was performed using the R software incorporating five clinical factors (age, performance status, elevated LDH level, B symptoms, and stage) and four TIME prognostic factors (T cell percentage, T to abnormal B cell ratio, normal B to abnormal B cell ratio, and NK cell percentage) which were significant in univariate analyses (**Supplementary Figure 3A**). LASSO regression analysis returned five independent risk factors after dimensionality reduction. Subsequent multivariate Cox regression analysis determined the hazard ratios, and four prognostic factors were significant: the ratio of normal B cells to abnormal B cells, age, performance status, and presence of B symptoms (**Table 4**). A nomogram was built as a tool to predict 1-, 3-, and 5-year OS risk probability according to the model. The calibration curve showed good agreement between the model predictions and actual observed risk at 1-, 3-, and 5-years. The area under the curve (AUC) of time-dependent ROC curves for the generated Cox model for 1-, 3-, and 5-year OS risk prediction was 0.878, 0.855, and 0.936 respectively, which indicated the good discriminative ability of this model. Decision curve analysis results using the R software also suggested that this nomogram can be a valuable tool for decision making by clinicians (**Supplementary Figures 3B–E**).

**Table 4:** Model established by LASSO-Cox regression analysis in the study cohort.

Prediction factor	HR (95% CI)	*P*
NK cells in all cells ≤0.81%	1.32 (0.58, 3.04)	0.509
Ratio of normal B cells to abnormal B cells ≤0.61	8.44 (1.07, 66.81)	**0.043**
ECOG performance status >1	5.53 (2.70, 11.31)	**<0.0001**
Age >60 y	4.53 (1.82, 11.29)	**0.001**
Presence of B symptoms	2.67 (1.24, 5.74)	**0.012**

HR, hazard ratio; CI, confidence interval; ECOG, Eastern Cooperative Oncology Group. Significant *P* values are in bold.

**Figure 4:**
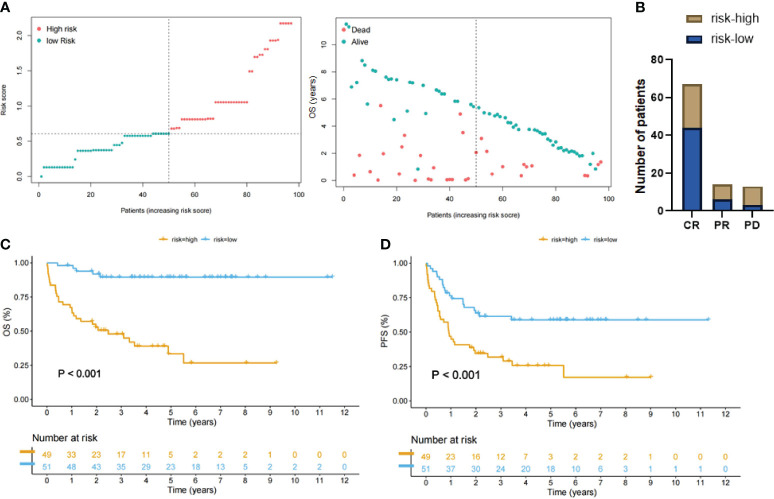
Patient groups stratified by LASSO-Cox regression analysis. **(A)** Left: Distribution of the patients with high-risk score (red color) and low-risk score (green color). Right: Scatter plot showing distribution of OS duration (years) of dead/alive patients according to the last follow-up data. **(B)** DLBCL patients with low-risk score had a higher complete remission rate compared with the high-risk group. **(C, D)** Kaplan-Meier curves showing that patients with high-risk scores (yellow lines) had significantly poorer overall survival (OS) and progression-free survival (PFS) than patients with low-risk scores (blue lines).

The LASSO-Cox model generated risk scores for 100 patients with 9-factor data available. We used the median risk score to divide the entire study cohort into high- and low-risk groups (**Figure 4A**). The low-risk group exhibited more favorable therapeutic responses, with an 83.0% proportion of complete remission cases, which was significantly higher than the 56.1% in the higher-risk group (**Figure 4B**). Moreover, Kaplan-Meier survival curves illustrated that patients in the high-risk group exhibited a markedly poorer OS (*P <*0.001) and significantly shorter PFS (**Figures 4C, D**).

## Discussion

The current study used available clinical flow cytometry data for DLBCL diagnosis to assess the prognostic role of abnormal and normal B cells, T cells, macrophages, NK cells, and granulocytes in DLBCL. Overall, the results confirmed that abundant TILs (normal B, T, and NK cells) indicated favorable clinical outcomes. Granulocyte percentages with a very high cutoff (>50%) showed an unfavorable prognostic effect only in univariate but not multivariate analysis. The prognostic significance of T cells, the most abundant cell population in the cell suspension detected by flow cytometry, has been revealed by several previous flow cytometry studies in retrospective cohorts of DLBCL patients (31–34). In addition, our results demonstrated that the abundance of normal B cells had significantly favorable prognostic effect in overall cohort and in patients with high percentages of T cells in DLBCL, which has not been studied by previous flow cytometry studies, and validated our recent findings in a different DLBCL cohort through ultra-deep immunoglobulin sequencing of DNAs extracted from formalin-fixed paraffin-embedded (FFPE) tissues (19). These results suggest that clinical flow cytometry data can be used for prognostic prediction in addition to diagnosis. The clinical utility of TIME features assessed by flow cytometry was highlighted by the prognostic Lasso-Cox model and the nomogram constructed as a predictive tool in this study; however, we should be aware of the limitations of the predictive model and quantification of cell populations by flow cytometry, as the quantification can vary with different gating strategies and between different laboratories (35), and the cutoffs and prognostic results in this retrospective cohort need be validated by larger-scale studies.

Flow cytometry and IHC are complementary clinical practices that are routinely used for the diagnosis of hematologic neoplasms. A recent study demonstrated that high immunoscores based on CD3 and CD20 IHC staining, routinely performed in the clinic, were significantly associated with favorable clinical outcome (36); however, routine IHC could not distinguish between normal and abnormal B cells. Compared to IHC, flow cytometry uses larger fresh tissues and is more sensitive to detect selected marker expression (37). However, previous studies have consistently shown that DLBCL cell populations can be missed in flow cytometry results, likely caused by the tissue disaggregation process, fibrosis, cell fragility, necrosis, and sample size, and diagnosis-failure cases are often those with high non-lymphocyte non-granulocyte content, high T cell proportion among lymphocytes, and low tumor MYC expression (27, 38–41). Likely due to similar reasons and the low frequency of normal B cells, we found that normal B cells reached prognostic significance when they were evaluated by their ratios to abnormal B cells or by their frequencies in all B cells in DLBCLs, but not by their percentages in all viable cells, the evaluation method for T/NK cells. Using ratios of different B cell populations might reduce laboratory variations due to differences in gating. Compared with the results by our previous study which estimated normal B cell frequencies through immunoglobulin sequencing in a multi-center *de novo* DLBCL cohort (19), the mean and median ratios of normal to abnormal B cells by phenotyping in this flow cytometry cohort were comparable: 0.35/0.048 by flow cytometry phenotypic analysis vs. 0.44/0.049 by immunoglobulin genetic differentiation (mean/median ratios of normal B-cell clonotype counts to malignant B-cell clonotype counts). The mean/median frequencies of normal B cells among all B cells, another method to evaluate normal B cells in DLBCL, were 15.06%/4.6% by flow cytometry and 14.17%/4.64% by immunoglobulin clonotype analysis (19).

Compared with our previous results by fluorescent mIHC on FFPE tissues (18), which method has the advantage of quantifying immunophenotypic marker co-expressions in a spatially resolved manner, the mean and median percentages of T cells among all viable cells by flow cytometry were significantly higher (41%/42.75% vs. 24.7%/19.4%), whereas the median percentage of total B cells (41.24% vs. 52.1%) and mean/median percentages of monocytes (1.18%/0 vs. 8.8%/5. 1% macrophages) among all viable cells by flow cytometry were significantly lower. These differences may stem from the two different analysis methods. Fluorescent mIHC imaging analysis often selects tumor cell-enriched regions of interest for quantification, whereas flow cytometry uses fresh cell suspensions from larger biopsy tissues, whose cellular constitution is affected by the tissue disaggregation process however. Similarly, the state-of-the-art single-cell RNA sequencing (scRNA-seq), which also relies on single-cell suspension generated by tissue disaggregation, has shown technical issues leading to misleading definition of macrophage subpopulations (42). Nonetheless, scRNA-seq data have revealed the intra- and inter-tumor heterogeneity of TME in lymphoma (43, 44), which can be relevant for the response to anti-cancer drugs and residual disease characterization (45, 46). However, scRNA-seq comes with higher costs in terms of reagents, equipment, and manpower for sequencing and analysis of each individual cell (47). Thus, scRNA-seq studies tend to be small to moderate scale and are potentially prone to dissociation distortions and patient-specific heterogeneity (43, 48). In contrast to these limitations of scRNA-seq, multicolor flow cytometry can quantify immune and clonal tumor cells more cost-effectively and expeditiously, enabling correlative analysis in a large-scale cohort, and has great potential for evaluating lymphoma TIME with implications for tailoring personalized immunotherapy regimens for patients (49), as demonstrated by the nomogram and decision curve analysis in this study.

The prognostic cutoff for high normal B-cell abundance in the current flow cytometry analysis was much higher than an optimal cutoff used in our previous immunoglobulin clonotype study (19). Possible causes may include the methods used to define normal B cell populations, inclusion of different DLBCL subtypes and treatments in this small study cohort, tumor heterogeneity and tissue selection, as well as functional heterogeneity of normal B cells affecting the prognostic effect (50, 51). Flow cytometry isolates cell clusters using different antibody combinations (such as CD5/CD19, CD10/CD19, CD20/CD138, CD38/CD56, etc. in this study) and abnormal B cell clusters are identified by light chain restriction (monotypic kappa or lambda). Non-restricted (polytypic) B cell clusters are viewed as normal B cells (for some cases without a specific percentage reported, differences between total B and abnormal B cell percentages were used in this study). However, clonal assessment by flow cytometry has potential technical issues such as precise gating, low sensitivity of light chain restriction analysis, and antibodies (52, 53). The normal kappa to lambda ratio range is generally 0.5 to 3.0 in literature (52, 54). In a recent scRNA-seq study in follicular lymphoma, the ratios of kappa to lambda of likely normal B cell clusters ranged from 1.38 to 2.57 (43). However normal B cell kappa/lambda light-chain expression can also be skewed (55). In a previous study by Roider et al. (45), light-chain-restricted clusters (defined as comprising >80% kappa+ or lambda+ B cells) were considered to also include normal B cell fractions, with the assumption that normal B cells have balanced kappa and lambda light chains. Altogether, the quantification of normal B cells by flow cytometry and phenotyping might be not accurate.

Our results underscore the important roles of TILs in DLBCL clinical outcome, and support immunotherapies utilizing T cells and NK cells (56–58). However, both CAR-T cells and CAR-NK cells, which have been successful or promising, face the issue of frequent relapses (57, 59), suggesting that a better understanding of anti-tumor immune responses is still needed. In our cohort, although overall the T cell percentage in all cells was highly prognostic, the cutoff had to be low (~10% at the bottom 11 percentile), and a higher T cell percentage had no further prognostic significance among the high T-cell percentage group. This prognostic pattern was also observed in our previous mIHC study in a different DLBCL cohort, which also found that high PD-1 expression in T cells was associated with significantly poorer survival in the T cell-positive patient group (18). Thus, the existence of T cell-related immunosuppressive mechanisms may explain the limited prognostic effects of T cell percentages. The prognostic cutoffs for T cells by flow cytometry in the literature were slightly higher than ours, including 20% and 14% cutoffs for low T cell proportion in two previous flow cytometry studies (31, 33) and a 23% median cutoff for percentage of CD4 T cells in another study (32). A recent study using digitally quantified CD3+ and CD20+ scores by chromogenic IHC on whole slides of DLBCL tissues adopted much higher cutoffs for CD3+ cells with significant prognostic effects in the studied 104 DLBCL cases: the cutoff for high CD3+ percentage in all cells was at the top 21 percentile, and the cutoff for high CD3+ to CD20+ ratio was at the top 47 percentile (36). In a previous study by Autio et al. (60), T cell proportion in 188 DLBCL patients (median, 16%) by fluorescent mIHC was comparable to that in our mIHC study (18), and high proportions of TIM3+, LAG3+, and/or PD-1+ T cells were associated with poor clinical outcome in one to two of the two DLBCL cohorts (60). Also in this study, “hot” vs. “cold” TME, defined according to expression of an immune cell transcriptional signature and consistent with T cell markers by mIHC, was not associated with significant differences in survival of DLBCL patients (60). In contrast, in an independent study using a same gene-expression profiling panel (NanoString Technologies), Leivonen et al. found that a T lymphocyte transcriptional gene signature was prognostic in DLBCL and PTL (cutoffs, at the bottom 17.7 percentile and 20 percentile, respectively) (61), and also confirmed the adverse prognostic effects of lower percentages of CD3+CD4+ and CD3+CD8+ TILs in PTL patients using mIHC (61).

Our comprehensive results for TIME components may also provide a different perspective to understand why more than a dozen randomized studies conducted over the past two decades have failed to further improve prognosis by adding novel agents to the frontline R-CHOP regimen (62). One of these agents, bortezomib, inhibits the numbers and functions of activated normal naïve B, memory B, plasma cells, and CD4 T cells, whereas it increases regulatory T cell numbers and enhances NK cell-mediated anti-tumor responses (63). Ibrutinib, a BTK inhibitor, inhibits T cell activation, stimulates IFN-γ production, enhances macrophage-mediated antibody-dependent effector function (64, 65), and potentially inhibits normal B cell proliferation (66).

In summary, flow cytometry data provides a systems-level view of the TIME in DLBCL, and the results showed significant prognostic values of TILs (normal B, T, and NK cells) in DLBCL, suggesting additional clinical utility of flow cytometry analysis. Differences in TIME data between flow cytometry and IHC or genetic analysis in DLBCL were also noted, suggesting potential caveats of single-cell data requiring tissue disaggregation. As immunotherapy has gained prominence in lymphoma treatment, we can envision that TIME evaluation will play a more important role in clinical practice and the treatment selection for DLBCL in the future.

## Data availability statement

The raw data supporting the conclusions of this article will be made available by the authors, without undue reservation.

## Ethics statement

The studies involving humans were approved by the Institutional Review Board of Duke University. The studies were conducted in accordance with the local legislation and institutional requirements. Exempt Category: Category 4: Secondary research for which consent is not required: Secondary research uses of identifiable private information or identifiable biospecimens, if at least one of the following criteria is met: i. The identifiable private information or identifiable biospecimens are publicly available; ii. Information, which may include information about biospecimens, is recorded by the investigator in such a manner that the identity of the human subjects cannot readily be ascertained directly or through identifiers linked to the subjects, the investigator does not contact the subjects, and the investigator will not re-identify subjects; iii. The research involves only information collection and analysis involving the investigator’s use of identifiable health information when that use is regulated under 45 CFR parts 160 and 164, subparts A and E [HIPAA], for the purposes of “health care operations” or “research” as those terms are defined at 45 CFR 164.501 or for “public health activities and purposes” as described under 45 CFR 164.512(b); or iv. The research is conducted by, or on behalf of, a Federal department or agency using government-generated or government-collected information obtained for nonresearch activities, if the research generates identifiable private information that is or will be maintained on information technology that is subject to and in compliance with section 208(b) of the E-Government Act of 2002, 44 U.S.C. 3501 note, if all of the identifiable private information collected, used, or generated as part of the activity will be maintained in systems of records subject to the Privacy Act of 1974, 5 U.S.C. 552a, and, if applicable, the information used in the research was collected subject to the Paperwork Reduction Act of 1995, 44 U.S.C. 3501 et seq. *This Declaration of Exemption from further IRB Review is in effect from October 24, 2019 and does not expire. However, changes to the proposed research will require an amendment requesting re-review for exemption. Reportable serious adverse events and unanticipated problems related to the research that place subjects or others at risk of physical, psychological, economic, or social.

## Author contributions

TY: Data curation, Formal analysis, Investigation, Methodology, Software, Visualization, Writing – original draft. ZX-M: Conceptualization, Data curation, Formal analysis, Investigation, Methodology, Validation, Visualization, Writing – original draft. AL: Methodology, Resources, Writing – review & editing. WS: Methodology, Resources, Writing – review & editing. BW: Methodology, Resources, Writing – review & editing. JN: Methodology, Resources, Writing – review & editing. LC: Methodology, Resources, Visualization, Writing – review & editing. EC: Methodology, Resources, Writing – review & editing. SP-O: Methodology, Resources, Writing – review & editing. KY: Conceptualization, Data curation, Funding acquisition, Methodology, Project administration, Resources, Supervision, Writing – review & editing.
