# Supplementary material for: Flow cytometry quantification of tumor-infiltrating lymphocytes to predict the survival of patients with diffuse large B-cell lymphoma
Source: Front Immunol. 2024 Jan 29;15:1335689. doi: 10.3389/fimmu.2024.1335689 (PMC10859492; doi:10.3389/fimmu.2024.1335689)

Supplementary Figure legends

Figure S1. Flow cytometry analysis of immune cells in the DLBCL cohort. (**A**) Dot plots for normal B cells in the example DLBCL case in Figure 1. The isolated CD10-negative/CD19+ cell cluster (dark green) showing polytypic light chain restriction were considered as normal B-cells. (**B**) Correlation matrix for abundance of granulocytes, monocytes, NK cells and normal B cells. (**C**) High ratios of CD5+ T cells to abnormal B cells were associated with significantly better survival in the sub-cohort of patients with high T cell percentages in cells obtained from DLBCL tissues.

Figure S2. Kaplan–Meier survival analysis. (**A**) The favorable prognostic effect of high normal B cells (high ratios to abnormal B cells or high percentage in all B cells) shown in overall cohort in Figure 2C remained significant after the exclusion of non-treated DLBCL patients (left panel) and did not depend on the effect by T cell percentage (middle panel). The effect remained significant with further exclusion of patients treated with R-EPOCH regimen at frontline (right panel. Further restriction analysis in patients treated with upfront R-CHOP only showed similar results, but differences between groups 1 and 2 became non-significant $P = 0.090$ likely due to the smaller case numbers). (**B-C**) Upfront treatment with R-CHOP regimen and receiving hematopoietic stem cell transplantation (HSCT) were associated with significantly better clinical outcome in the overall cohort. (**D**) CAR-T therapy in the refractory/relapsed setting was associated with significantly better survival of patients with refractory/relapsed DLBCL.

Figure S3. LASSO (least absolute shrinkage and selection operator)-Cox regression analysis. (**A**) The LASSO coefficient profiles for nine factors identified by univariate analysis by tuning lambda parameter (λ). A 10-fold cross-validation plot based on the minimum criteria was used to select the optimal tuning parameter (λ). Dotted vertical lines were placed at the minimum criteria value and at a point representing one standard error above the minimum criteria. (**B**) The Area Under Curve (AUC) of receiver operating characteristics curves indicating good prognostic performance of the constructed prognostic model. (**C**) Nomogram using five predictors to predict 1-, 3- and 5-year OS event risk of DLBCL patients. Red points illustrate the use of the nomogram for an example case. The sum of five variables′ points is 0.729 for this case as shown on the total score axis, and the corresponding 1 year-, 3 year- and 5 year-risk probability is aligned below. A vertical red line from the point of 0.729 gives the risk probability values and 95% CI ranges. Abbreviations: PS, performance status; Pr, probability; futime, follow-up time. (**D**) Calibration curves for the predicted 1 year-, 3 year- and 5 year-risk probability by the nomogram and the actual observation. (**E**) Decision curve analysis for the nomogram. The y-axis measures net benefit. The x-axis shows a continuum of potential risk thresholds. The three horizontal gray lines are for the assumption of no intervention, the red, green, purple colored lines marked with All-1, 3, 5 are for the assumption of intervention for all patients at 1, 3, 5 years, and the black/dark navy blue, orange, blue colored lines marked with LR (Lasso regression)-1, 3, 5 are for the nomogram risk prediction. The greater positive net benefits indicate that this nomogram exhibited superiority than the “no intervention” or “intervention for all” options within a large probability threshold range at 1-, 3-, and 5-years, respectively.

Suppl. Figure 1

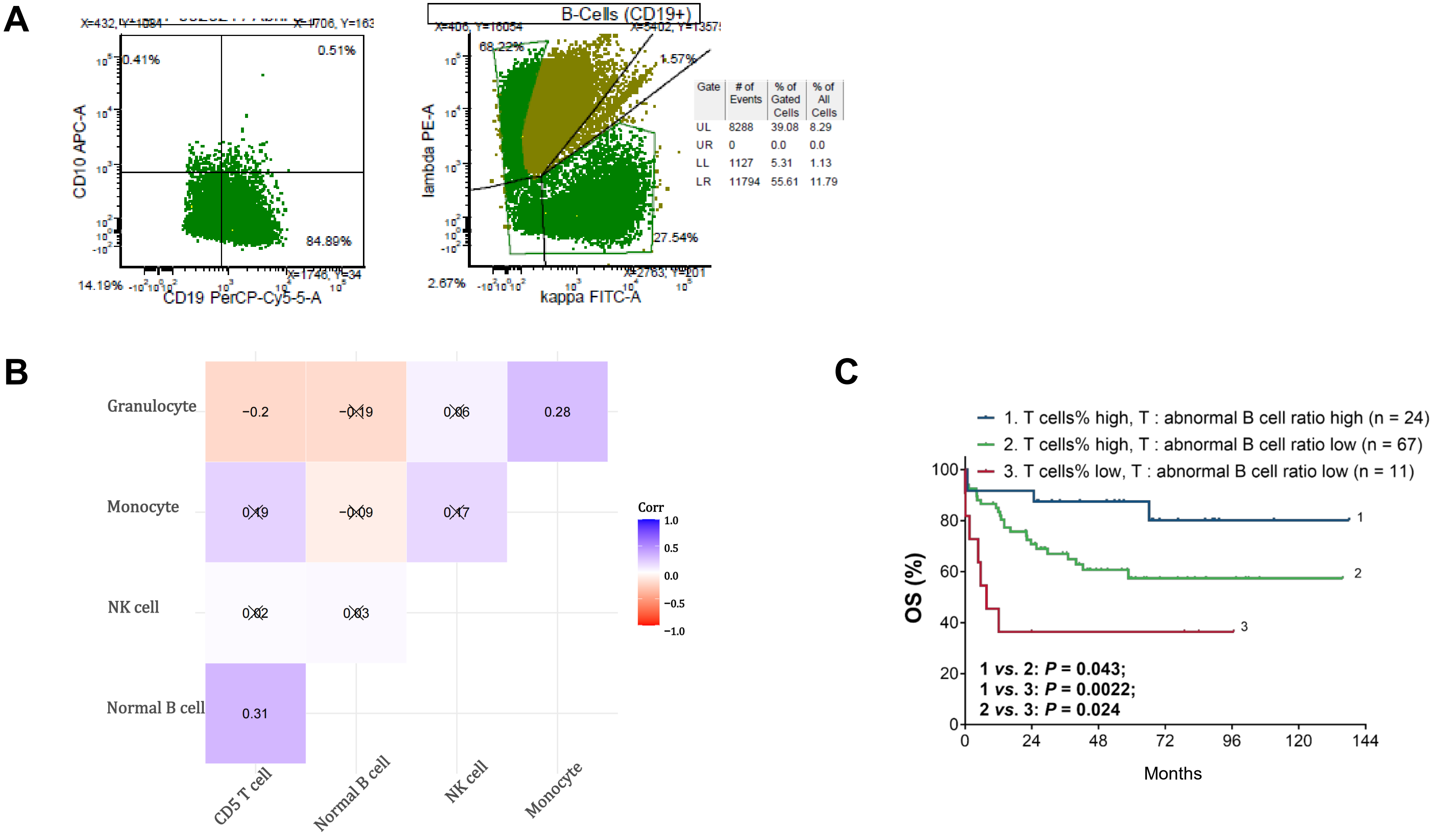

Suppl. Figure 2

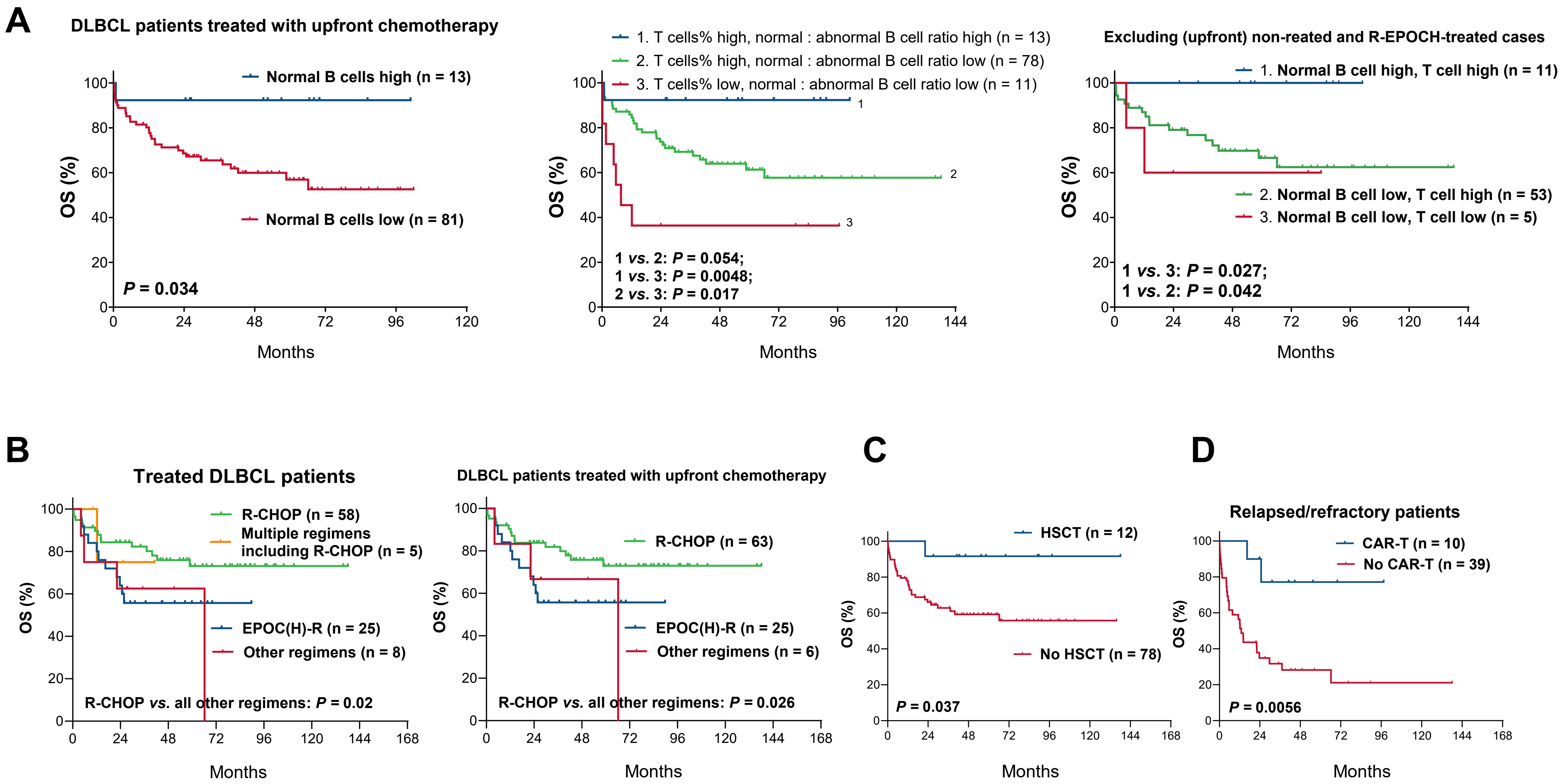

Suppl. Figure 3

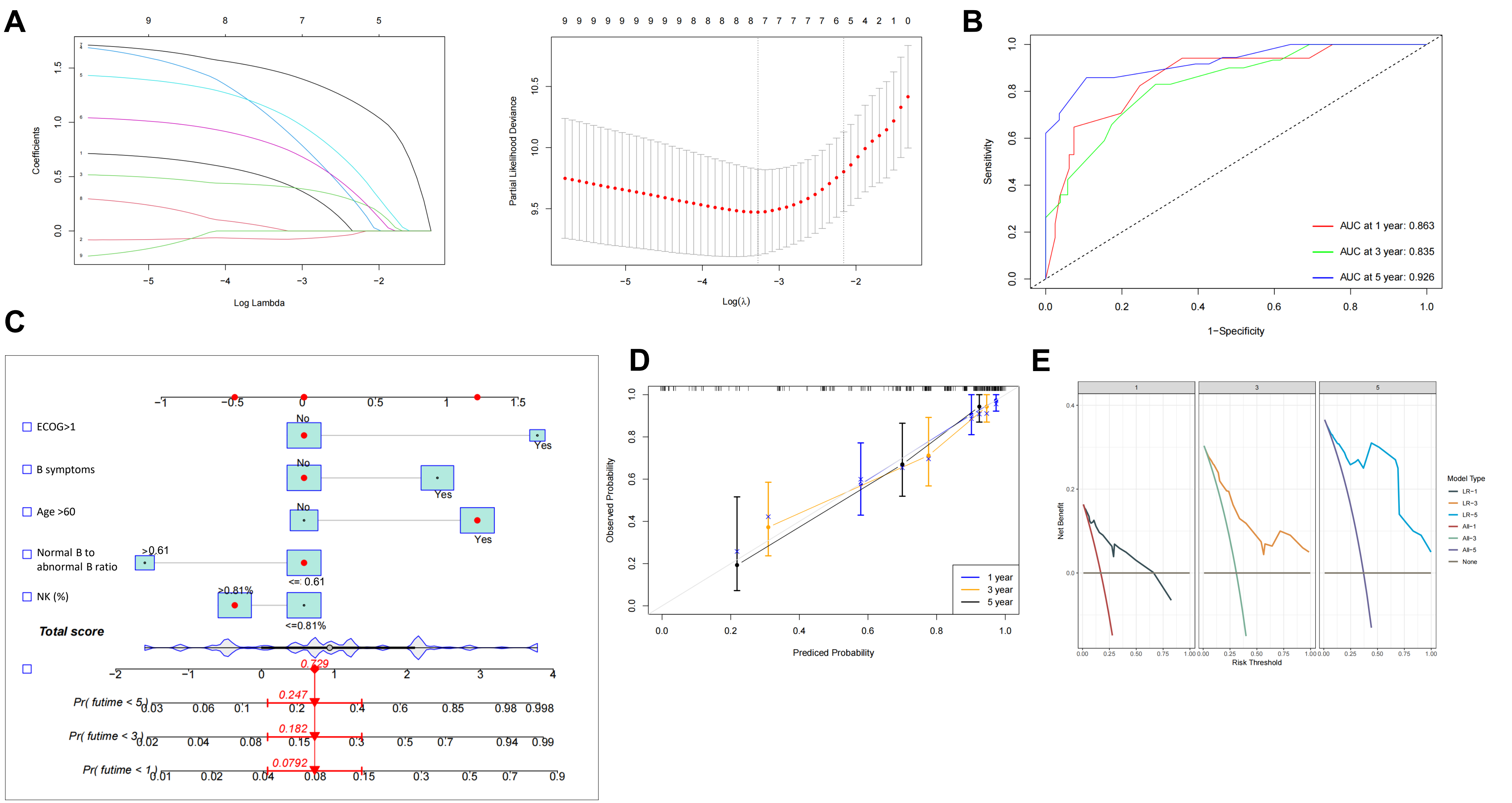

Supplement: Supplementary file 1 [file DataSheet_1.pdf]
